# Large Absorption Enhancement in Ultrathin Solar Cells Patterned by Metallic Nanocavity Arrays

**DOI:** 10.1038/srep34219

**Published:** 2016-10-05

**Authors:** Wei Wang, Jiasen Zhang, Xiaozhou Che, Guogang Qin

**Affiliations:** 1 State Key Lab for Mesoscopic Physics, School of Physics, Peking University, Beijing 100871, China; 2 Collaborative Innovation Center of Quantum Matter, Beijing, 100871, China

## Abstract

A new type of light trapping structure utilizing ring-shaped metallic nanocavity arrays is proposed for the absorption enhancement in ultrathin solar cells with few photonic waveguide modes. Dozens of times of broadband absorption enhancement in the spectral range of 700 to 1100 nm is demonstrated in an ultrathin Si_3_N_4_/c-Si/Ag prototype solar cell by means of finite-difference time-domain (FDTD) simulation, and this dramatic absorption enhancement can be attributed to the excitation of plasmonic cavity modes in these nanocavity arrays. The cavity modes optimally compensate for the lack of resonances in the longer wavelength range for ultrathin solar cells, and eventually a maximum *J*_*sc*_ enhancement factor of 2.15 is achieved under AM 1.5G solar illumination. This study opens a new perspective for light management in thin film solar cells and other optoelectronic devices.

Photovoltaics, the conversion of sunlight into electricity, is a promising technology which can generate electrical power on a very large scale without causing pollutions. Though growth in installed solar capacity has been very large over the past decades, the overall cost per watt of solar electricity is still one of the main obstacles to make it to meet a major part of the increasing energy demand in modern society^1^. Thus, the cost of solar electricity still need to be reduced definitely, which requires great advances in both cost reduction and efficiency improvement.

Until now, more than 86% of the solar cell market is still based on crystalline silicon wafers^2^, which account for around 24% of the total cost of the corresponding solar modules^3^. With much less material consumption, lower manufacturing cost and reasonable efficiency, thin film solar cell technology has attracted a great deal of research to reduce the cost per watt of solar electricity over the past decades^4^. However, a limitation in many thin-film solar cell technologies that based on collection-limited materials is the tradeoff between sufficient light absorption and efficient carrier collection, which limits the attainable solar cell efficiencies. In recent years, nanostructured light trapping schemes have emerged as a promising route towards improved efficiency in thin film solar cells^5^,^6^,^7^,^8^,^9^. A variety of deliberate designs of plasmonic^10^,^11^,^12^,^13^,^14^,^15^,^16^,^17^,^18^,^19^ and photonic^20^,^21^,^22^,^23^,^24^,^25^,^26^,^27^,^28^ nanostructures have been proposed, which can couple and bind light into photovoltaic active regions efficiently, providing ideal building blocks for the realization of novel and efficient solar cells with much thinner cell thickness. Especially, if the cell thickness can be reduced to be comparable to the minority carrier diffusion length, e.g. about 100 nm for a-Si:H, most collection-limited materials would benefit from ultrathin film devices with optically thick absorption.

However, solar cells with thicknesses comparable to minority carrier diffusion lengths usually support few waveguide modes, especially for longer wavelengths. Thus, light trapping schemes that couple light into these waveguide modes would perform badly due to lack of resonance. An alternative approach to increase resonance is to exploit surface plasmon polaritons (SPPs), which are electromagnetic surface waves confined to a metal-dielectric interface by coupling to the free electron plasma in metals^29^,^30^,^31^. Since in many thin film solar cells, the photovoltaic active layer is adjacent to the corresponding metal contact, e.g. polymer solar cells, it is convenient to incorporate plasmonic nanostructures on the metal contacts to manipulate SPPs on the metal-dielectric interface. Thus, the evanescent nature and strong field enhancement of SPPs can be sufficiently employed, making it possible to greatly improve the poor absorption of ultrathin solar cells suffering from deficient photonic waveguide mode resonance.

In this work, a new type of light trapping scheme employing ring-shaped plasmonic nanocavity^32^ arrays is proposed, in which the incident light flux is turned by 90° and efficiently absorbed along the lateral direction of the ultrathin solar cell. The launched SPPs on the metal-dielectric interface are tuned into various cavity modes by the nanocavity array, which can optimally compensate for the lack of waveguide mode resonances for longer wavelengths. Thus, dozens of times of absorption enhancement in this spectral region can be achieved. The absorption enhancement mechanism and corresponding resonant responses are systematically studied via electromagnetic simulations, which has been proved to be a critical and viable tool for optimizing the design of photonic nanostructures with good fidelity^19^. Moreover, the simulation routine facilitates the identification of different resonances together with knowledge on their origin, which enables a more thoughtful and effective optimization of the overall absorption of sunlight^9^.

## Results

**Figure 1:**
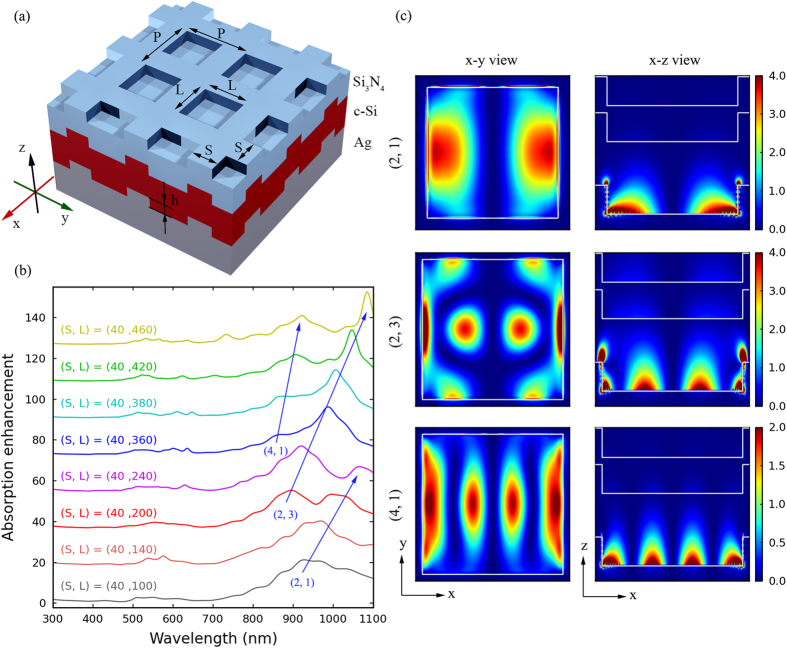
(**a**) Schematic of the simulated structure. The ring-shaped nanocavities on the Ag bottom layer are arranged in a square lattice, with lattice constant *P*, side length *L*, depth *h* and ridge width *S* = *P* − *L*. Then, the subsequent 100 nm c-Si and 50 nm Si_3_N_4_ layers are textured conformally, forming the absorption enhancement design. (**b**) Absorption enhancement for nanocavity arrays with fixed *S* = 40 nm and different *L*’s as a function of wavelength, relative to the corresponding bare structure. These curves are offset for clarity. (**c**) Patterns for |E_z_|^2^ component of the electric field in the x-y plane 10 nm above the bottom of the Ag nanocavity and in the x-z plane at the center of the array unit cell for various cavity modes, in which material profiles are marked by white lines.

Figure 1a illustrates the general absorption enhancement design for prototype solar cells which consists of three layers, Ag bottom layer, 100 nm thick c-Si and 50 nm thick Si_3_N_4_. This structure represents a typical problem in ultrathin solar cells lacking in waveguide mode resonance, since the 100 nm thick c-Si layer here supports only the lowest order photonic waveguide mode in the wavelength range of 700 to 1100 nm. The c-Si is used, since it is the most prevalent photovoltaic material as well as the material system considered in many photonic and plasmonic solar cell designs^7^,^9^,^10^,^13^,^14^,^15^,^18^,^20^,^21^,^23^,^24^,^26^,^28^. Additionally, the Si_3_N_4_ layer acts as an optimized antireflection coating for the corresponding bare Si_3_N_4_/c-Si/Ag structure. The ring-shaped plasmonic nanocavity array is patterned on the Ag bottom layer, and the subsequent c-Si and Si_3_N_4_ layers are textured conformally. The nanocavity array consists of square nanocavities with side length *L*, depth *h* and lattice constant *P*, which are arranged in a square lattice. The nanocavities are separated by ridges with width *S*, and obviously have *S *= *P* − *L*. In the simulations, for simplicity, the cavity depth *h* is fixed to 40 nm, a value slightly smaller than half the c-Si layer thickness.

Firstly, a series of nanocavity arrays with fixed ridge width (*S *= 40 nm) and varying cavity side lengths (*L*) ranging from 100 to 460 nm are simulated, and the corresponding absorption spectra of the c-Si layer are shown in Fig. 1b, which are normalized to that of the corresponding optimized bare structure. In this figure, several times of absorption enhancement can be found in the shorter wavelength range of 300 to 700 nm, but the more remarkable result is the broadband dramatic absorption enhancement (dozens of times) in the longer wavelength range of 700 to 1100 nm, where the 100 nm thick c-Si layer supports only the lowest order photonic waveguide mode. Additionally, these large absorption enhancement peaks can be classified into several red shifting series as indicated by the blue arrows, since peaks of the same series have similar electric field spatial distributions which correspond to the same plasmonic cavity mode. These red shifting cavity mode resonances can be understood by a simple analytical model, in which the resonant condition for a specific normal mode (m, n) of the nanocavity is given by^32^


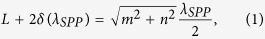


where *λ*_SPP_ is the SPP wavelength and *δ*(*λ*_SPP_) the penetration depth of SPPs into the Ag reflectors, which depends on the phase shift of plasmon reflection at the reflectors, the size of the cavity, and the height of the reflectors. Mode profiles (|*E*_z_|^2^ component of the electric field) for the (2, 1), (2, 3), and (4, 1) modes in a unit cell of the nanocavity arrays are plotted in Fig. 1c, and the corresponding (*L*, cavity mode resonant wavelength) are (240, 1064) nm, (420, 1048) nm, and (420, 908) nm, respectively. The x-y view of the mode profiles is on the x-y plane 10 nm above the bottom of the nanocavity, and the x-z view on the x-z plane at the center of the array unit cell. In these plots, the cavity regions in the x-y view and the cell profiles in the x-z view are marked by white lines. From these mode profiles it can be seen that, with exponentially decaying electric fields, the SPP standing waves in these cavity modes are optimally bound within the ultrathin c-Si layer of the solar cell, and the electric fields are greatly enhanced in the c-Si layer region. Therefore, activation of these cavity modes can dramatically enhance absorption of the c-Si layer in the longer wavelength region.

**Figure 2:**
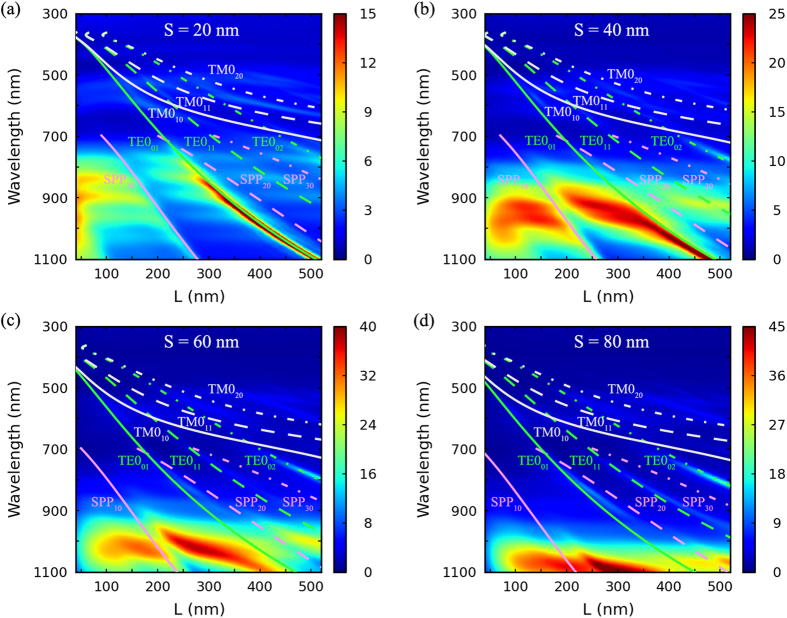
(**a**–**d**) Absorption enhancement for nanocavity arrays as a function of wavelength and cavity side length *L* for different ridge width *S*’s, relative to the corresponding bare structure. As a reference, dispersion curves of SPP, TM, and TE waveguide modes that possibly exist in the nanocavity arrays under different phase matching conditions are marked in the graphs, respectively.

In order to fully optimize the light trapping performance, the absorption spectra of the c-Si layer in the nanocavity arrays with various ridge widths (*S *= 20, 40, 60, and 80 nm) and continuously varying cavity side lengths (*L*) ranging from 40 nm to 520 nm are calculated, and normalized to that of the corresponding bare structure as well. Thus, the absorption enhancement factors as a function of cavity side length (*L*) and illumination wavelength for various ridge widths are obtained and shown in Fig. 2, in which an ultra-small step of 4 nm for *L* is chosen in the optimization.

Notably, dozens of times of broadband absorption enhancements are observed in the spectral range of 700 to 1100 nm in this figure, which can be attributed to the excitation of plasmonic cavity modes in these nanocavity arrays. To make a reference, the numerically calculated dispersion curves of various waveguide modes that possibly exist in the nanocavity arrays are overlaid on the maps in Fig. 2, according to the following phase matching conditions,


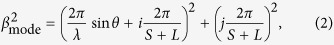


where *i*, *j *= 0, ±1, ±2…, denoting different phase matching conditions, *λ* the incident wavelength, *θ* the incident angle and *β*_mode_ is the propagation constant of a specific waveguide mode, for which the corresponding value in the bare structure is used for simplicity. To distinguish different waveguide modes under various phase matching conditions that travel in different directions, a specific notation, mode_ij_, is used, in which mode_i0_ travels along x direction, mode_0j_ along y direction, and mode_11_ along the diagonal of x and y direction. Thus, the absorption enhancement patterns in the spectral range of 700 to 1100 nm can be divided into several regions by the SPP_10_, TE0_01_, TE0_11_, SPP_20_, SPP_30_, and TE_02_ dispersion curves. Then, the spatial distribution of |*E*_z_|^2^ component for each of the absorption enhancement peaks in the above spectral range for the nanocavity arrays with *L* ranging from 40 to 520 nm with a step of 20 nm is checked. It is found that, in the region near and on the left side of the SPP_10_ curve, all of the absorption enhancement peaks have electric field mode patterns as the (2, 1) cavity mode pattern shown in Fig. 1c, and this region is recognized as the (2, 1) cavity mode region. In the region near the TE0_01_ curve and between the TE0_01_ and SPP_10_ curves, the absorption enhancement peaks originate from the (2, 3) cavity mode shown in Fig. 1c, and this region is recognized as the (2, 3) cavity mode region. The region near SPP_20_ curve is recognized as the (4, 1) cavity mode region. Additionally, higher orders of cavity modes can be found on the right side of this region. Thus, the great absorption enhancement in the spectral range of 700 to 1100 nm can be attributed to the excitation of plasmonic cavity modes in these nanocavity arrays.

**Figure 3:**
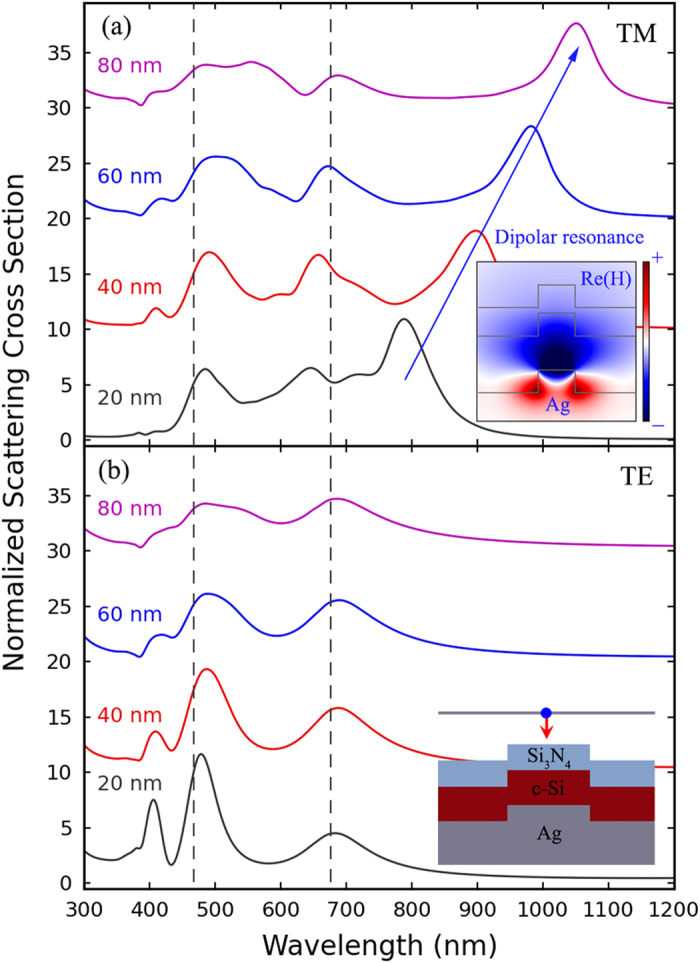
Scattering cross-sections for various ridges with different width *S*’s, normalized to the corresponding geometrical cross-section, under (**a**) TM and (**b**) TE broadband normal incident plane wave illumination. These curves are offset for clarity. The blue arrow indicates the red-shifting dipolar resonance when *S* increases. Inset of panel (a) shows Re(*H*) field profile for the *S* = 60 nm wide ridge under dipolar resonance, and inset of panel (b) shows a schematic for the ridge.

The absorption enhancement peaks show red-shifting behaviors when the cavity side length *L* increases, which can be explained by the cavity mode resonance condition described by equation (1). For small *S*, the cavity modes can couple with the TE0_01_ and SPP_10_ waveguide modes, as a result, the absorption enhancement ribbons stretch along the red-shifting dispersion curves, which can be seen in Fig. 2a,b. Additionally, the absorption enhancement factor of the cavity modes increases with *S*, which can be attributed to the increase of the launching efficiency of the cavity modes. To further illustrate this phenomenon, scattering properties of the ridges are calculated using 2D FDTD method. In the simulations, a TM or TE polarized plane wave source is normally incident upon the ridges (the inset of Fig. 3b) and the ridge width *S* is changed. The corresponding scattering cross sections normalized to the geometrical cross sections, *S*, are plotted in Fig. 3a,b. The peaks indicated by dashed lines can be attributed to Fabry-Pérot resonance in the multilayered thin film structure, which do not depend on *S*. The peaks indicated by blue arrow in Fig. 3a originate from the localized surface plasmon (LSP) resonance, which correspond to similar magnetic field profiles as shown in the inset of Fig. 3a, and do not show in Fig. 3b for the TE incident. When *S* increases, the localized resonance on the ridges will have a larger space to reside in, thus the corresponding resonant mode redshifts. Then, by comparing the red-shifting phenomenon of the horizontal strip-like patterns in Fig. 2 to that of the localized resonance in the ridges when *S* increases from 40 nm to 80 nm, it can be concluded that, the activation of cavity modes corresponding to the horizontal strip-like patterns mainly depends on the resonant scattering of the ridges between the nanocavities. Additionally, as increasing *S* can increase effective scattering cross sections, as well as the launching efficiency of SPPs, thus launching efficiency of the cavity modes increases, and then larger absorption enhancements can be observed for longer wavelengths.

Finally, in order to quantify the absorption enhancement of different nanocavity arrays across the solar spectrum, the maximum short circuit current density is calculated by assuming that each absorbed photon with energy greater than the band gap of c-Si produces one and only one electron-hole pair. Then, the photo-generated carriers are perfectly collected, giving the maximum short circuit current density,


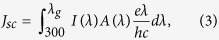


where *λ* is the wavelength, *I*(*λ*) the spectral irradiance (power density) of the ASTM AM 1.5G solar spectrum^33^, *A*(*λ*) the absorptance, and *λ*_g_ the wavelength corresponding to the band gap of c-Si. The evaluation of the integral is done by using the trapezoid method in the range of 300 to 1100 nm, approximately the above bandgap absorption bandwidth of c-Si.

**Figure 4:**
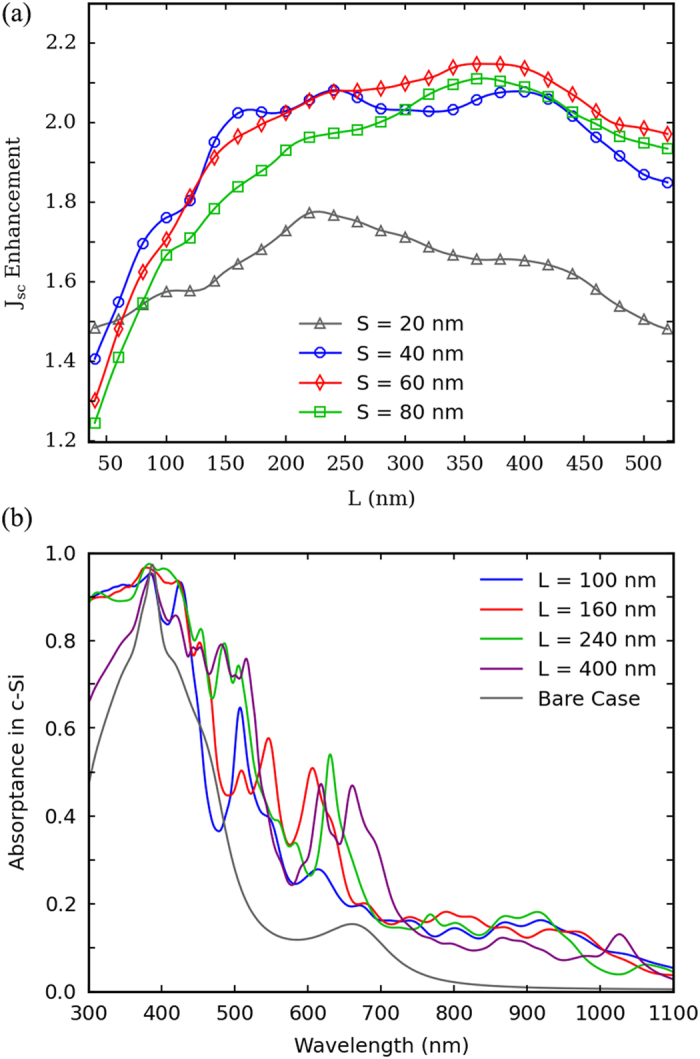
(**a**) *J*_*sc*_ enhancement as a function of cavity side length *L* for nanocavity arrays with various ridge width *S*’s, relative to the corresponding bare structure. (**b**) Absorption spectra for nanocavity arrays with the same *S (S* = 40 nm) but different *L*’s, and the case for the corresponding bare structure is also shown.

The *J*_*sc*_’s for different *L*’s and *S*’s are calculated and normalized to that of the corresponding bare structure, thus the *J*_*sc*_ enhancement factors are obtained, as show in Fig. 4a. It can be seen that nanocavity arrays with *S* = 40, 60 and 80 nm have similar *J*_*sc*_ enhancement curves and all of them can greatly improve the integrated absorption. While, for *S* = 80 nm, the resonant wavelength of the LSP mode is larger than 1000 nm, the non-resonance results in a relatively lower absorption enhancement effect in the spectral range of 850 to 1000 nm, as well as a lower *J*_*sc*_ enhancement. Then, an optimized design is obtained at *S* = 60 nm and *L* = 360 nm, corresponding to a *J*_*sc*_ enhancement factor of 2.15. Due to small scattering cross section, nanocavity arrays with *S* = 20 nm have poor *J*_*sc*_ enhancement performance.

Additionally, as can be noticed, there are several local maxima or critical points in the *J*_*sc*_ enhancement curves in Fig. 4a. For nanocavity arrays with *S* = 40, 60 and 80 nm, since the absorption enhancement exhibits horizontal strip-like patterns as mentioned above, cavity modes contributes about the same to the total integrated absorption when *L* varies. The difference in *J*_*sc*_ enhancement mainly originates from the difference in light coupling into waveguide modes for shorter wavelengths, as shown in Fig. 4b. In this graph, absorption spectra of c-Si for nanocavity arrays with the same *S (S* = 40 nm) but different *L (L* = 100, 160, 240, and 400 nm, which corresponds to a local maximum or a critical point in the *J*_*sc*_ enhancement curve) are plotted, and the absorption spectrum of c-Si layer in the corresponding bare structure is also curved as a reference.

**Figure 5:**
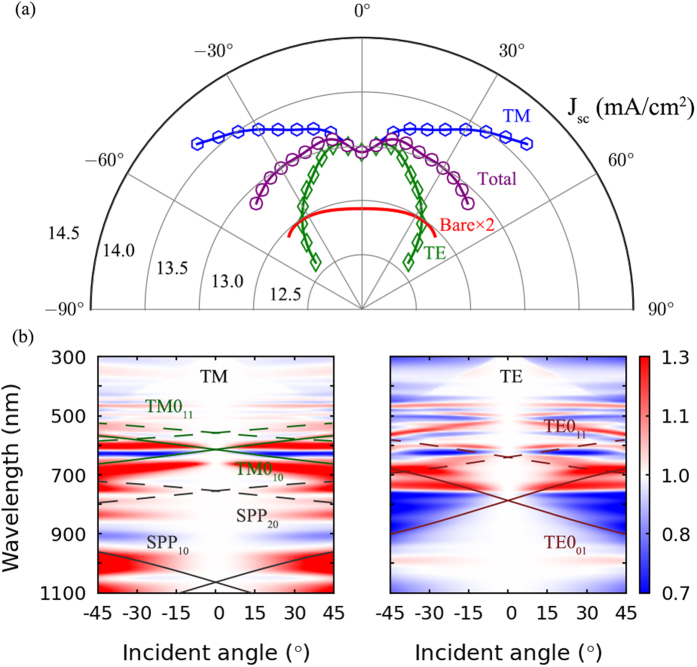
(**a**) Angular dependence of the maximum short circuit current *J*_*sc*_ for an *S* = 40 nm and *L* = 240 nm nanocavity array. The TM and TE polarization averged *J*_*sc*_ for the corresponding bare structure is also marked in the figure, after doubled. (**b**) The corresponding absorption spectra under TM and TE polarization conditions, normalized to that under normal incidence. The overlaid lines on the map are dispersion curves for the lowest order waveguide modes that possibly exist in the nanocavity arrays under different phase matching conditions.

To illustrate the angular dependence of absorption enhancement of the nanocavity arrays, an optimal nanocavity array with *S* = 40 nm and *L* = 240 nm is specifically studied. The corresponding maximum short circuit current *J*_*sc*_ as a function of incident angle under TM, TE and Total (unpolarized) polarization conditions is calculated respectively and shown in Fig. 5a. Additionally, the Total *J*_*sc*_ for the corresponding bare three-layered thin film structure, which is doubled before plot for easy comparison, is also marked in the figure. It can be seen that, different angle dependent properties of *J*_*sc*_ are observed under TM and TE polarizations when the incident angle increases from 5 to 45 degrees, i.e. the *J*_*sc*_ for TM polarization increases when the incident angle become larger, while the *J*_*sc*_ for TE polarization decreases. Therefore, the total effect of TM and TE polarization is nearly angle insensitive, with a *J*_*sc*_ enhancement factor maintaining at least a factor of two compared to the corresponding bare structure. Additionally, the corresponding absorptance spectra, normalized to that under normal incidence, as a function of incident angle under TM and TE polarization conditions are shown in Fig. 5b, which shows how the absorptance spectra change with incident angle. And dispersion curves for the lowest order waveguide modes that possibly exist in the nanocavity arrays under different phase matching conditions are also overlaid on the maps, as in Fig. 2. It can be seen that, due to the TM polarized nature of the SPP modes, when the incident angle increases, coupling between the incident light and the nanocavity modes will become stronger under TM polarization condition, and the coupling will become weaker under TE polarization condition due to the shading effect of the cavity walls. That’s the reason for the opposite angle dependent properties of the absorption enhancement under TM and TE polarization conditions, resulting in a nearly angle insensitive absorption enhancement property of the nanocavity arrays under unpolarized incident light.

## Discussion

In the above design and simulations, a new type of light trapping scheme utilizing ring-shaped plasmonic nanocavity arrays for the absorption enhancement in ultrathin solar cells is proposed, and the absorption enhancement in ultrathin 50 nm Si_3_N_4_/100 nm c-Si/Ag prototype solar cells patterned by various nanocavity arrays is specifically studied using FDTD simulation. Plasmonic cavity modes in the nanocavities can dramatically improve the light absorption in the longer spectral range of 700 to 1100 nm where c-Si has fairly low absorption coefficients. For the spectral range of 300 to 700 nm, absorption enhancement can be attributed to light coupled into waveguide modes by the nanocavity arrays. Using an optimal array configuration of *S* = 60 nm and *L* = 360 nm, a maximum *J*_*sc*_ enhancement factor of 2.15 can be achieved under AM 1.5G solar illumination. Additionally, a good angle insensitive enhancement property is also demonstrated on the nanocavity arrays. Therefore, metallic nanocavity arrays can be a useful design for the light-trapping in solar cells, photo-detectors and other optoelectronic devices with metallic back contacts, in which the bottleneck arising from the balance between efficient absorption and limited device thickness troubles.

## Methods

The optical properties of Ag, c-Si, and Si_3_N_4_ are taken from experimental measurements^34^, and fitted to a multi-coefficient material model^35^ before simulation. Broadband illumination (spectral range 300–1100 nm, approximately the above bandgap absorption bandwidth of crystalline silicon) is simulated via a plane wave source above the nanocavity arrays with electric field polarized along the x-axis. For structures with such small dimensions, wave effects must be taken into account by solving the full wave vector Maxwell’s equations. Therefore, three-dimensional broadband finite-difference time-domain (FDTD) simulations are employed to calculate the absorption within the ultrathin c-Si layer in the nanocavity arrays. The electric fields are recorded in the range of 300–1100 nm with a wavelength step of 4 nm, then the absorption per unit volume (*P*_*abs*_) due to material absorption is calculated from the divergence of the Poyting vector^35^


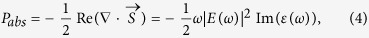


where |*E*(*ω*)|^2^ is the electric field intensity squared and *ε*(*ω*) the corresponding material dielectric function. During the simulation, several tricks are employed to guarantee a high accuracy of the absorption results. Firstly, since during FDTD simulations each field component (*E*_*x*_, *E*_*y*_, *E*_*z*_) is calculated at a different location within the Yee cell^36^, *P*_*abs*_ corresponding to each field component is calculated first and then interpolated back to a common set of points (the origin of the Yee cell) before adding together. This method avoids errors arising from difference of *ε*(*ω*) at different locations within the Yee cell. Secondly, an ultrafine mesh is overlaid on the nanocavity region with a mesh size of 2 nm, by which every feature size of the simulated structure can be divisible. This avoids errors arising from the cross-interface mesh cells. Lastly, since only absorption that occurs in the c-Si layer contributes to the photocurrent of the solar cell, absorption in the c-Si layer must be isolated from that within other layers. Given the refractive index on the interface is different from that of the adjacent materials in the FDTD algorithm, spatial position rather than refractive index is used to distinguish different materials before applying volume integration on *P*_*abs*_ in the c-Si region. Thus, a high accuracy c-Si absorption spectrum is obtained, which can match perfectly with that obtained using the transfer matrix method (TMM, of which results can be regarded as the analytical solution) simulation for the corresponding multilayered bare structure.

Finally, as the incident angle of sunlight can derive from the surface normal, the angular dependence of absorption enhancement for the nanocavity arrays is calculated. Since angle dependent FDTD simulations usually convergence much slower than the normally incident cases, nanocavity arrays with large lattice constants may be computationally too time intensive to give satisfactory angular dependent results. Therefore, a relatively smaller nanocavity array with *S *= 40 nm and *L *= 240 nm is chosen, which corresponds to a locally optimized maximum short circuit current shown in Fig. 4a, to investigate the angular dependence of the absorption enhancement. TM, with electric field in the incident plane, and TE, with electric field perpendicular to the incident plane, polarization conditions are simulated, respectively. Then the unpolarized results, which are denoted as “Total” in Fig. 5a, are obtained by simply averaging the corresponding results under TM and TE polarization conditions.

## Additional Information

**How to cite this article**: Wang, W. *et al.* Large Absorption Enhancement in Ultrathin Solar Cells Patterned by Metallic Nanocavity Arrays. *Sci. Rep.* **6**, 34219; doi: 10.1038/srep34219 (2016).

## Supplementary Material

Supplementary Information
