# Supplementary material for: Large Absorption Enhancement in Ultrathin Solar Cells Patterned by Metallic Nanocavity Arrays
Source: Sci Rep. 2016 Oct 5;6:34219. doi: 10.1038/srep34219 (PMC5050426; doi:10.1038/srep34219)
Supplement: Supplementary Information [file srep34219-s1.doc]

**Large Absorption Enhancement in Ultrathin Solar Cells Patterned by Metallic Nanocavity Arrays**

**Wei Wang^1^, Jiasen Zhang^1,2,*^, Xiaozhou Che^1^ and Guogang Qin^1,*^**

^1^State Key Lab for Mesoscopic Physics, School of Physics, Peking University, Beijing 100871, China

^2^Collaborative Innovation Center of Quantum Matter, Beijing, 100871, China

*Authors to whom correspondence should be addressed. Electronic addresses: qingg@pku.edu.cn and jszhang@pku.edu.cn


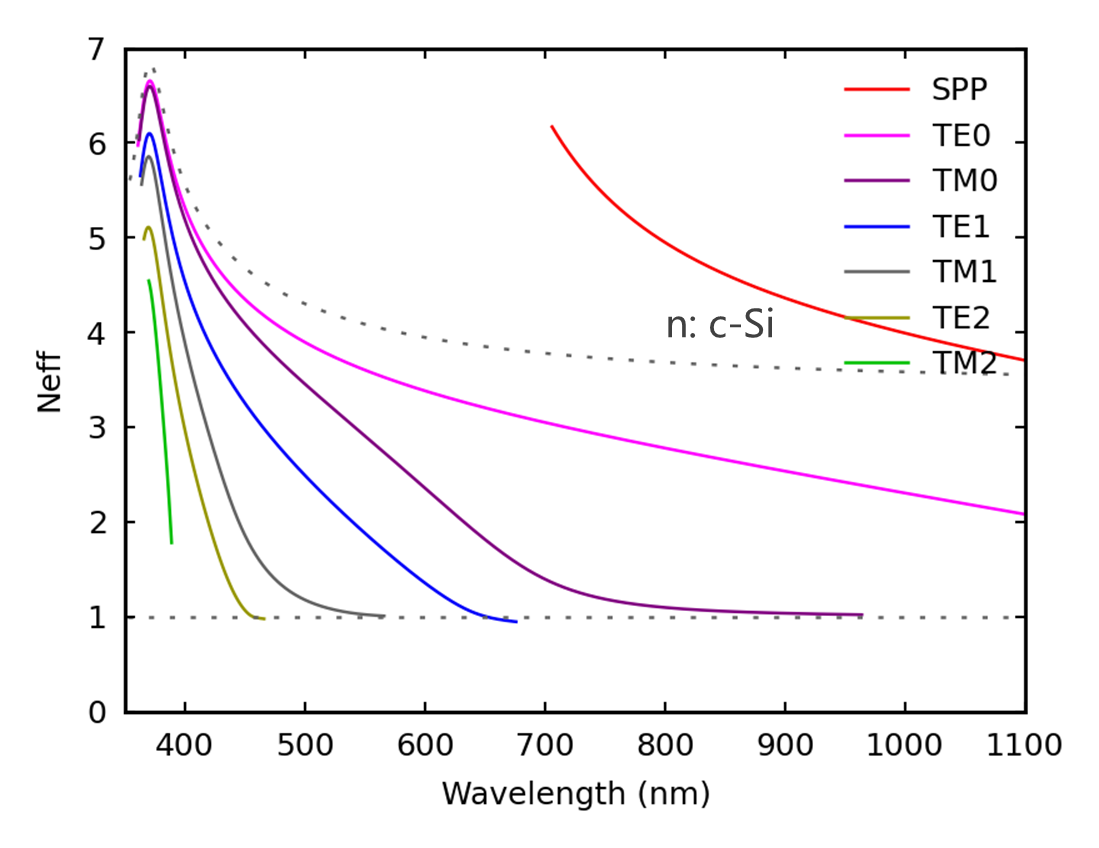


**Figure S1.** Numerically calculated dispersion curves for effective indexes of the lowest seven order waveguide modes that exist in the bare 50 nm Si_3_N_4_/100 nm c-Si/Ag multi-layered structure. To make a reference, refractive indexes of Air (n=1) and c-Si are marked by gray dotted lines in the graph, respectively.


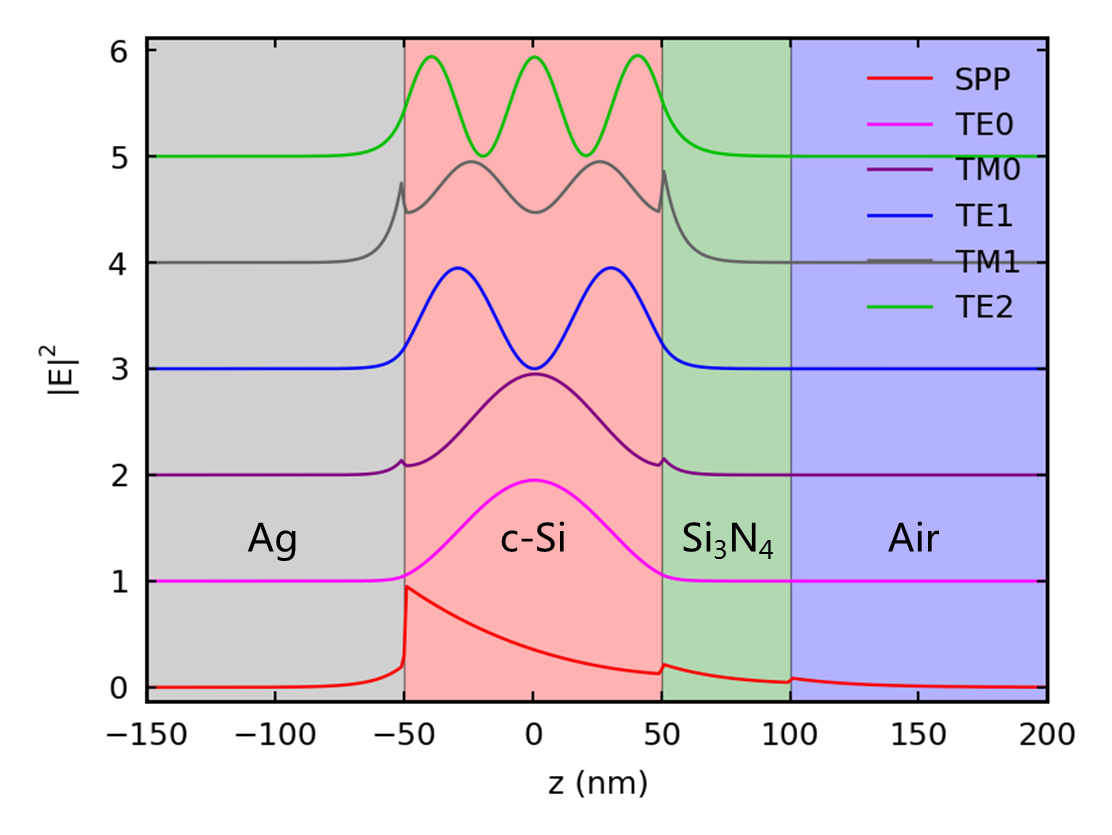


**Figure S2.** Normalized electric field intensity profiles for various waveguide modes that exist in the bare 50 nm Si_3_N_4_/100 nm c-Si/Ag multi-layered structure, which are offset for clarity. The corresponding vacuum wavelength for the SPP mode is 1100 nm and 380 nm for the others.
